# Learning from a hybrid programme delivering presbyopia services in India

**Published:** 2026-03-12

**Authors:** Rollo Romig, Miranda Pursley, Amit Gupta

**Affiliations:** 1 Manager: Solutions Insights Lab, Brooklyn, USA.; 2 Impact Research Manager: Solutions Insights Lab, Brooklyn, USA.; 3 COO: The/Nudge Institute, Bangalore, India.

**A pilot project has already generated helpful insights that could be replicated in other countries that want to address presbyopia.**

**Figure:**
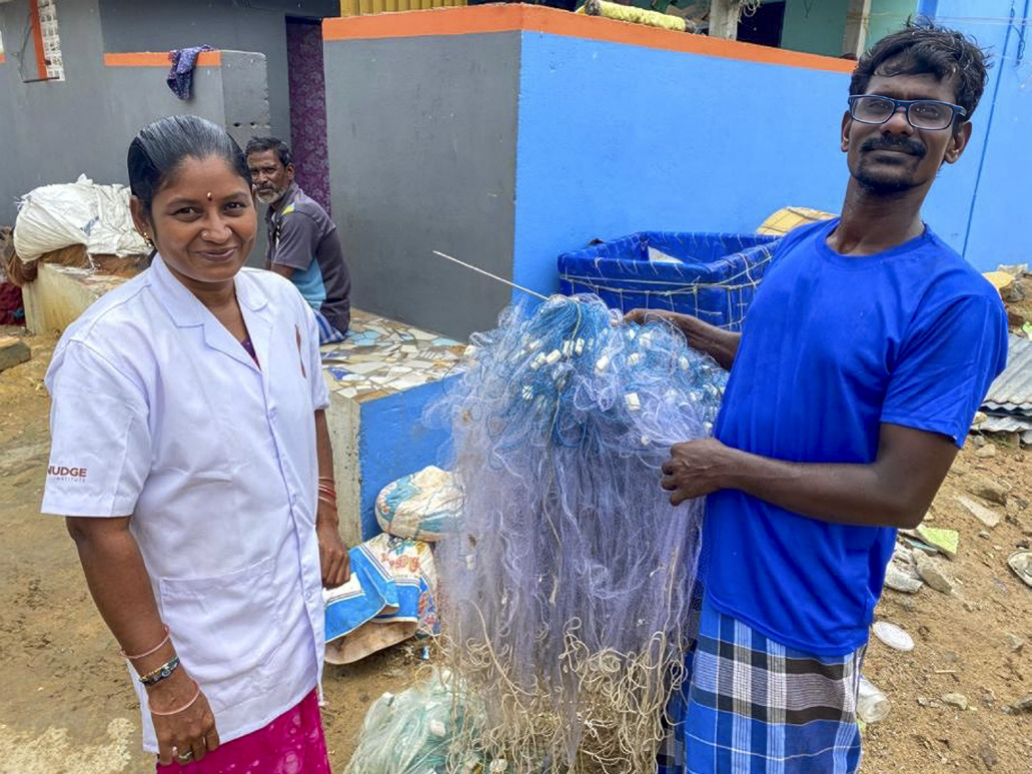
Padmavati, an entrepreneur, with Mani, a fisherman who bought near-vision spectacles from her. INDIA

© ROLLO ROMIG CC BY-NC-SA 4.0

“After getting positive feedback from one fisherman, I sold around 20 pairs of reading glasses to 20 other fishermen,” said Padmavati, an eyeglass entrepreneur in Tamil Nadu. ‘Reading glasses,’ it turns out, is a misnomer. A better term is ‘near-vision spectacles,’ because it captures all the many reasons why people rely on them. And for middle-aged fishermen, near-vision spectacles are invaluable for repairing their nets. This was exactly what Padmavati had been hoping to see: a single demonstration cascading into many more sales. But it was also a revelation. Who knew that fishermen wanted near-vision spectacles?

Nearly everyone 40 years and older is affected by presbyopia, which makes it hard to focus on near objects. However, up to 826 million people worldwide^1^ lack the affordable, simple spectacles that would correct it, which has a profound effect on their livelihoods. A huge number of those who need near-vision spectacles - some 300 million people - is concentrated in India. Here, The/Nudge Institute, a poverty-alleviation organisation, is implementing a model that works with local entrepreneurs and the established system of community health workers in the country to distribute ready-made near-vision spectacles to people who do not have access to vision centres, whether because of cost, distance, or both. In the long term, the hope is that increased awareness of presbyopia, and the uptake of near-vision spectacles, will grow a sustainable market that will benefit existing vision centres.

Community health workers and entrepreneurs are supported to go into the community to find people who would benefit from near-vision spectacles. As part of their training, they also learn how to identify other eye conditions and refer customers to an eye care worker (such as an optometrist) if they suspect they are seeing something other than presbyopia.

The pilot stage of the project has already generated helpful insights that could be replicated in other countries that want to address presbyopia.

1. **Connect to existing networks.** When it comes to public health interventions, India's great advantage is its array of community networks, both grassroots and government-led. In the northeastern state of Meghalaya, for example, community health workers are actively collaborating with local village health councils when setting up free eye screening and near-vision spectacle distribution at vision camps. And in Tamil Nadu, entrepreneurs are spreading the word through women's self-help groups.
2. **Learn from entrepreneurs.** Because entrepreneurs must convince people in order to make a sale, they must “test pitches, timing, product choices, and price points,” said Ankur Sanghai, who leads The/Nudge's entrepreneur programme. This generates real-time, actionable feedback that can be incorporated into standard operating procedures. For example, in Tamil Nadu, entrepreneurs learned that many customers actually prefer bifocals, to avoid the hassle of taking their near-vision spectacles on and off as needed.
3. **Demand is about visibility and trust, not just price.** “There isn't as much lack of awareness around the problem as we think there is,” said Amit Gupta, COO of The/Nudge. “The lack of awareness is regarding how cheap the solution is.” In Karnataka, about 90% of recipients were still using near-vision spectacles months later - and were willing to pay an average of INR 350, or around USD 4, for a second pair. In short, once people trust the product, modest payment is sustainable.
4. **Demonstrate immediate impact and build trust.** The entrepreneurs have learned to increase impact by offering immediate, customised demonstrations of the spectacles’ usefulness. If the potential customer is a cook, for example, the entrepreneur is ready with a tray of mixed lentils to show how much easier they are to sort when wearing near-vision spectacles. Offering group screening increases impact too: as one entrepreneur observed, “If one person buys the spectacles, another person will feel that they can also buy them, because it's trustworthy.”
5. **Policies and reframing can unlock scale.** Because near-vision spectacles are available over-the-counter in India, anyone can legally dispense them; this is a regulatory condition that lowers costs and opens non-medical channels. More important, reframing presbyopia as a livelihood issue opens up additional funding possibilities from livelihood and poverty alleviation programmes. That reframing may be the most powerful insight: near-vision spectacles can spread much further if we think of them not only as a health intervention, but also as a tool for earning.

## Notes

These insights were derived by Solutions Insights Lab, through interviews with The/Nudge Institute and other stakeholders involved in the project, including entrepreneurs, government stakeholders, and the people who received near-vision spectacles in the pilot stage. Read more about the project here:

bit.ly/4r8gqps

To read more insights into how other organisations are distributing near-vision spectacles, visit

bit.ly/3qTyBzt
